# New Perspectives in Dried Blood Spot Biomarkers for Lysosomal Storage Diseases

**DOI:** 10.3390/ijms241210177

**Published:** 2023-06-15

**Authors:** Justyna Spiewak, Ivan Doykov, Apostolos Papandreou, Jenny Hällqvist, Philippa Mills, Peter T. Clayton, Paul Gissen, Kevin Mills, Wendy E. Heywood

**Affiliations:** 1 Inborn Errors of Metabolism Section, Genetics & Genomic Medicine Programme, UCL Great Ormond Street Institute of Child Health, University College London, London WC1 1EH, UK; j.spiewak@ucl.ac.uk (J.S.); i.doykov@ucl.ac.uk (I.D.); apostolos.papandreou@alumni.ucl.ac.uk (A.P.); j.hallqvist@ucl.ac.uk (J.H.); p.mills@ucl.ac.uk (P.M.); peter.clayton@ucl.ac.uk (P.T.C.); p.gissen@ucl.ac.uk (P.G.); 2 Molecular Neurosciences, Developmental Neurosciences Programme, UCL Great Ormond Street Institute of Child Health, University College London, London WC1 1EH, UK; 3 Department of Neurology, Great Ormond Street Hospital for Children, London WC1N 3JH, UK; 4 Department of Metabolic Medicine, Great Ormond Street Hospital for Children, London WC1N 3JH, UK

**Keywords:** Fabry disease, glycosphingolipid, biomarker, Gaucher disease, mucopolysaccharidoses, Niemann–Pick C disease, dried blood spot

## Abstract

Dried blood spots (DBSs) biomarkers are convenient for monitoring for specific lysosomal storage diseases (LSDs), but they could have relevance for other LSDs. To determine the specificity and utility of glycosphingolipidoses biomarkers against other LSDs, we applied a multiplexed lipid liquid chromatography tandem mass spectrometry assay to a DBS cohort of healthy controls (*n* = 10) and Gaucher (*n* = 4), Fabry (*n* = 10), Pompe (*n* = 2), mucopolysaccharidosis types I–VI (*n* = 52), and Niemann–Pick disease type C (NPC) (*n* = 5) patients. We observed no complete disease specificity for any of the markers tested. However, comparison among the different LSDs highlighted new applications and perspectives of the existing biomarkers. We observed elevations in glucosylceramide isoforms in the NPC and Gaucher patients relative to the controls. In NPC, there was a greater proportion of C24 isoforms, giving a specificity of 96–97% for NPC, higher than 92% for the NPC biomarker N-palmitoyl-O-phosphocholineserine ratio to lyso-sphingomyelin. We also observed significantly elevated levels of lyso-dihexosylceramide in Gaucher and Fabry disease as well as elevated lyso-globotriaosylceramide (Lyso-Gb3) in Gaucher disease and the neuronopathic forms of Mucopolysaccharidoses. In conclusion, DBS glucosylceramide isoform profiling has increased the specificity for the detection of NPC, thereby improving diagnostic accuracy. Low levels of lyso-lipids can be observed in other LSDs, which may have implications in their disease pathogenesis.

## 1. Introduction

In the post-COVID-19 pandemic healthcare setting, there is growing demand for remote patient management. Dried blood spots (DBSs) can offer this convenience, and DBS enzyme and biomarker assays are proving increasingly useful in the diagnosis and monitoring of patients with lysosomal storage diseases (LSDs) [1,2,3,4]. Biomarker analysis can be performed in a multiplex manner, with many compounds extracted and measured at the same time. DBS collections are also less expensive, less invasive, associated with simplified sample storage and transport, and can be easily performed multiple times (even in a home setting); hence, they are very promising for the study and monitoring of neurometabolic conditions such as LSDs.

The glycosphingolipids (GSLs) are lipids that accumulate in the glycosphingolipidoses, but unusually for metabolic disorders, it is the surrogate deacylated lyso-GSL equivalent lipids that have been found to be more useful as biomarkers than the accumulated GSLs themselves [5,6]. What is interesting is that disruption of GSL degradation is implicated not just in the glycosphingolipidoses but in other non-glycosphingolipidoses LSDs [7,8]. The exact mechanism involved is not known. Many GSL biomarkers have been well characterised in the relevant disease, but little has been described about how these biomarkers present in other LSDs. This information could shed light on lysosomal function and the potential harmful effect the build-up of these metabolites may have on the cell beyond the main enzyme defect.

**Figure 1:**
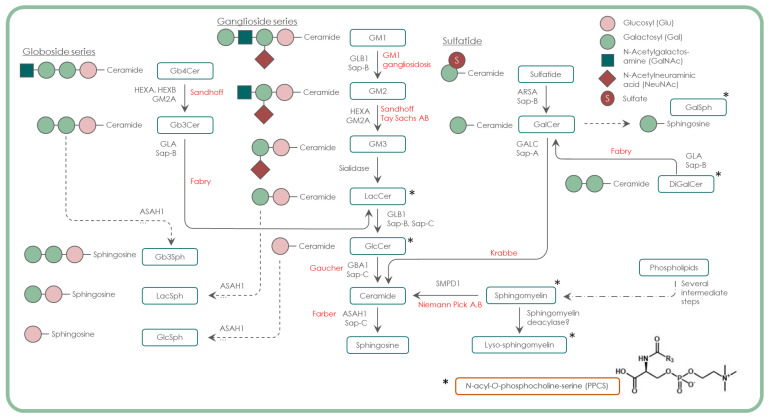
The glycosphingolipid degradation pathway, illustrating known enzyme defects and associated disorders (red text). Lipids that are included in the assay are indicated by *. PPCS presented individually, as the associated pathway is unknown.

We have previously described an optimised extraction protocol and ultra-high-performance liquid chromatography–tandem mass spectrometry (UPLC–MS/MS) method to measure 90 isoforms of 4 classes of GSLs from the glycosphingolipid degradation pathway (Figure 1) in DBS, as well as their “lyso” deacylated lipid forms [1]. This included the GSLs upstream and downstream of the GSL defects observed in each of the glycosphingolipidoses studied, to see if this would improve specificity for each individual disease and its specificity compared with other non-glycosphingolipidoses LSDs. We have also investigated the specificity of N-palmitoyl-O-phosphocholineserine (PPCS), previously known as lyso-sphingomyelin 509 [9], in a neurodevelopmental disease cohort. This biomarker has also been implicated in CLN3 disease, with a patient having been initially misdiagnosed as NPC due to PPCS elevation [10], thereby calling into question the specificity of this marker for LSDs.

Therefore, we aimed to determine the specificity of known biomarkers for Niemann–Pick type C (NPC), sphingomyelin (SM), and lyso-SM in an LSD disease cohort which included patients with mucopolysaccharidoses (MPS) and glycosphingolipidoses.

## 2. Results

### 2.1. Dried Blood Spot Glycosphingolipids in Lysosomal Storage Diseases

**Figure 2:**
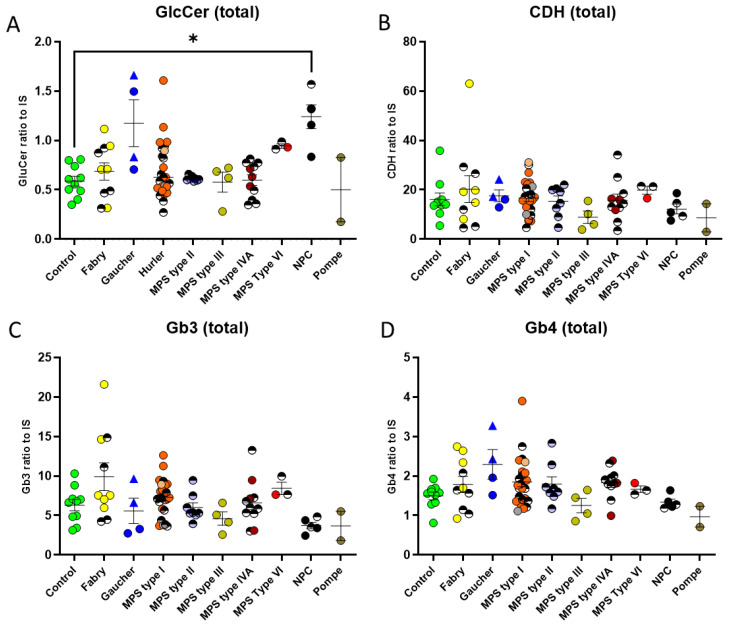
Dried blood spot glycosphingolipid levels in a cohort of patients with lysosomal storage diseases. The four main GSL species detectable in DBS are shown. (**A**) GlcCer—only significantly different to the controls for NPC, and therefore useful as a biomarker. (**B**) CDH—no significant changes observed. (**C**) Gb3 CDH—no significant changes observed. (**D**) GB4 CDH—no significant changes observed. Patients in treatment are represented by half shaded circles. Gaucher type I is represented by a triangle symbol, and type III, by a circle. The untreated MPS type I group is depicted as follows: Hurler in orange, Hurler–Scheie in grey, and Scheie in pink. Significance determined by Kruskal–Wallis non-parametric test. * means significance of *p* ≤ 0.05.

Glucosylceramide (GlcCer) is the main ceramide monohexoside (CMH) in blood and accumulates in the tissues and circulation of those with Gaucher disease. A total of 11 GlcCer isoforms with variable fatty acid chain lengths were included in our multiplex assay. GlcCer levels were measured in four Gaucher disease (GD) patients, two with type I GD and two with type III GD. We observed a median 2-fold elevation in the total GlcCer in the GD patients in comparison with the healthy controls (Figure 2A); however, two patients had levels within the control range. An assessment of the GlcCer levels in DBS from patients with other LSDs revealed that this GSL was also moderately increased in individuals with MPS VI (median-fold elevation of 1.6) and, as described previously [1], in NPC disease patients (approx. 2.3-fold). No statistically significant changes were seen for the other groups of LSDs when considered as a disease group, although 4 of the 10 Fabry and 6 of the 21 MPS I disease patients had levels slightly above the control range.

The ceramide dihexosides (CDHs), consisting of lactosylceramide (LacCer) and galabiosylceramide (Gb2), were assessed collectively, as reverse-phase chromatography does not separate structural isomers. Gb2 is a substrate of alpha-galactosidase A, the defective enzyme that results in Fabry disease (FD), and CDHs have been found previously to be elevated in FD patient urine [11,12]. However, we observed no significant differences in the total CDH (Figure 2B) for any of the disease groups, including that of Fabry, where only one patient, a 14-yr-old male, had elevated CDH levels (1.8-fold) above the highest control value.

Globotriaosylceramide (Gb3), the main GSL that accumulates in FD, was also assessed [13]. No significant increase was evident in any of the disease groups compared with the control DBS. Only three out of ten FD patients had levels higher than controls. Hence, DBS total Gb3 is not an ideal biomarker to monitor for Fabry disease (Figure 1C).

For globoside (Gb4), the molecule that cannot be broken down in Sandhoff disease (samples not available), we did not observe any significant disease group change. Certain patients within some of the disease groups did show higher levels of Gb4 than controls. Interestingly, these are the same patients who had high levels of GlcCer (Figure 2A).

**Figure 3:**
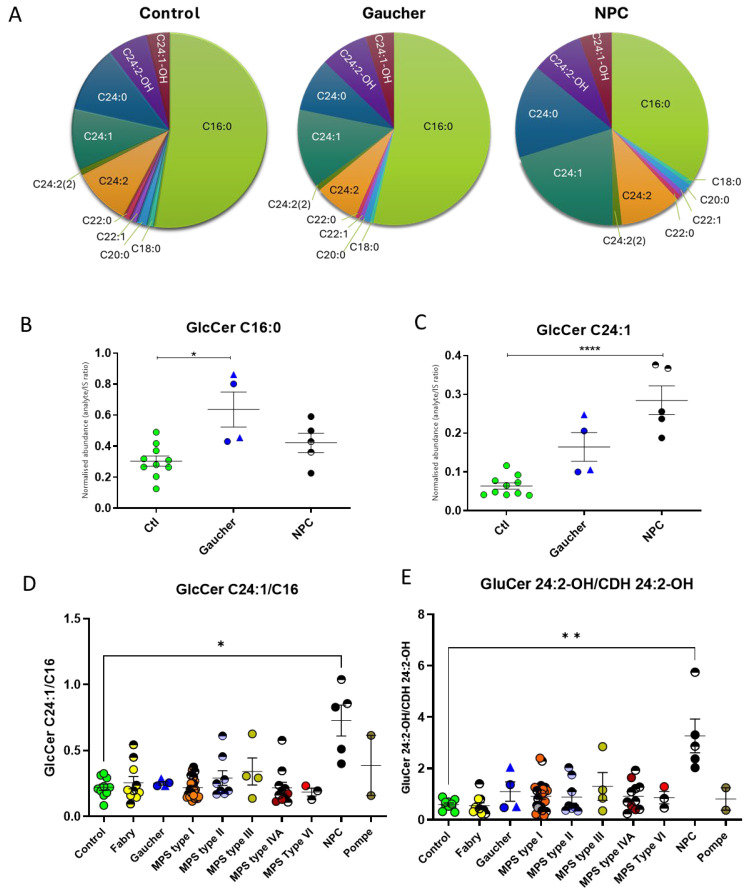
Glucosylceramide isoforms in dried blood spots from patients with lysosomal storage diseases. (**A**) A summary pie chart of the mean values of each isoform in the control, Gaucher, and NPC disease, showing a differing isoform profile in NPC disease. (**B**) The C16 isoform in Gaucher and NPC DBS in comparison with control levels. (**C**) The C24:1 isoform in Gaucher and NPC DBS in comparison with control levels. (**D**) Ratio of GlcCer C24:1/C16 isoforms in a cohort of patients with LSDs, showing that NPC has the greatest ratio difference relative to controls. (**E**) Ratio of GlcCer 24:2-OH/CDH 24:2-OH isoforms, showing the best specificity for NPC. Patients on treatment are represented by half-shaded circles. Gaucher type I is indicated by a triangle symbol, and type III, by a circle. The untreated MPS type I group circles are coloured as follows: Hurler as orange, Hurler–Scheie as grey, and Scheie as pink. **** means significance of *p* ≤ 0.0001; ** means significance of *p* ≤ 0.01; * means significance of *p* ≤ 0.05.

Although the total levels of each glycosphingolipid class for the various diseases proved informative, we also further evaluated the individual isoforms of GlcCer. In the controls and GD DBS, the C16:0 isoform accounts for more than 50% (Figure 3A). Relative to the control DBS, GD patients have an increase in the C16:0 isoform of GlcCer (Figure 3B). Surprisingly, the most dramatic change in the isoform profile for total GlcCer was observed for the NPC DBS (Figure 3A). NPC patients demonstrate a significant 4.4-fold increase in the C24 isoforms of GlcCer, with the most abundant being C24:1. Whilst there is also a 2.5-fold increase in this isoform in GD patients, this is not significant (Figure 2C). When we calculated the ratio of C24:1/C16, complete separation of NPC from both the control and GD samples was observed (Figure 3D).

### 2.2. Lyso-Glycosphingolipids in the Lysosomal Storage Diseases

Glucosylsphingosine and galactosylsphingosine (also known as psychosine) are derived from the CMH isoforms GlcCer and galactosylceramide, respectively, and are referred to collectively as lyso-CMH. Similarly to the CDH molecules, it is not possible to separate these isomeric lyso-molecules using reverse-phase chromatography. However, given that GlcCer is the predominant CMH in blood, it is expected that the predominant lyso-CMH in the DBS will be lyso-GlcCer, known as lyso-Gb1.

**Figure 4:**
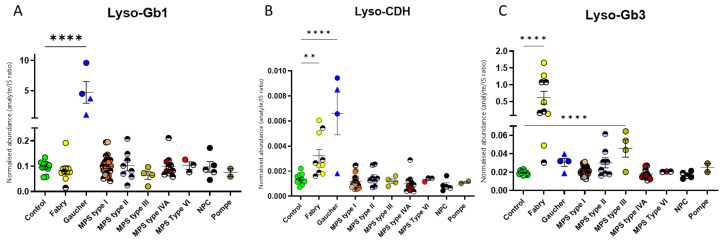
Lyso-glycosphingolipid dried-blood-spot analysis of samples from a cohort of patients with lysosomal storage diseases. (**A**) The Gaucher disease group has the highest levels of lyso-Gb1. Low-level changes in lyso-Gb1 in other LSDs were observed. (**B**) Small increases in the levels of lyso-CDH were seen in the Fabry and Gaucher samples. (**C**) The Fabry disease group has the highest lyso-Gb3 levels. Much smaller changes were observed for the other LSDs patients on treatment, represented by half-shaded circles. Gaucher type I samples are represented by a triangle symbol, and type III, by a circle. The untreated MPS type I group circles are coloured as follows: Hurler is orange, Hurler–Scheie is grey, and Scheie is pink. **** means significance of *p* ≤ 0.0001; ** means significance of *p* ≤ 0.01.

As expected, analysis of the DBS revealed lyso-Gb1 to be highly elevated in the GD group in comparison with control levels, showing a 41.1-median-fold change (Figure 4A). Overall, no large increases of lyso-Gb1 were seen in the other LSDs and noticeably not in NPC, even though NPC GlcCer levels were elevated. As with some of the GSLs, some individual patients within the FD, Hurler, MPS II, MPS VIA, and NPC disease groups displayed lyso-Gb1 levels higher than controls. Comparison of the lyso-Gb1 levels in these patients with their GlcCer levels revealed that only the FD patients had higher corresponding GlcCer levels. Interestingly, the other disease patients with the smaller lyso-Gb1 increases had GlcCer levels within the control range, indicating general lysosomal dysfunction may result in production of the toxic lyso-GSLs.

Lyso-CDH consists of lyso-lactosylceramide and lyso-galabiosylceramide, which derive from lactosylceramide and galabiosylceramide (Gb2), respectively. Both are present in blood. As mentioned previously, Gb2 is a substrate of alpha-galactosidase, and CDH accumulates in FD. Analysis of the total lyso-CDH revealed a significant increase in the median levels of lyso-CDH by 5.9- and 2.0-fold in GD and FD patients, respectively, when compared with healthy controls (Figure 4B).

Lyso-Gb3 is a well-established diagnostic biomarker for FD [14,15]. As expected, there was a large increase in the median levels (18.3-fold) of lyso-Gb3 in the FD patients when compared with the control group (Figure 4C). No other disease group demonstrated a large increase in lyso-Gb3 levels, although a small, significant 2.6-median-fold change was observed in the MPS III disease group, and two individuals from the MPS II group had levels above the control range (Figure 4C).

### 2.3. Dried Blood Spot NPC Biomarkers in Lysosomal Storage Diseases

**Figure 5:**
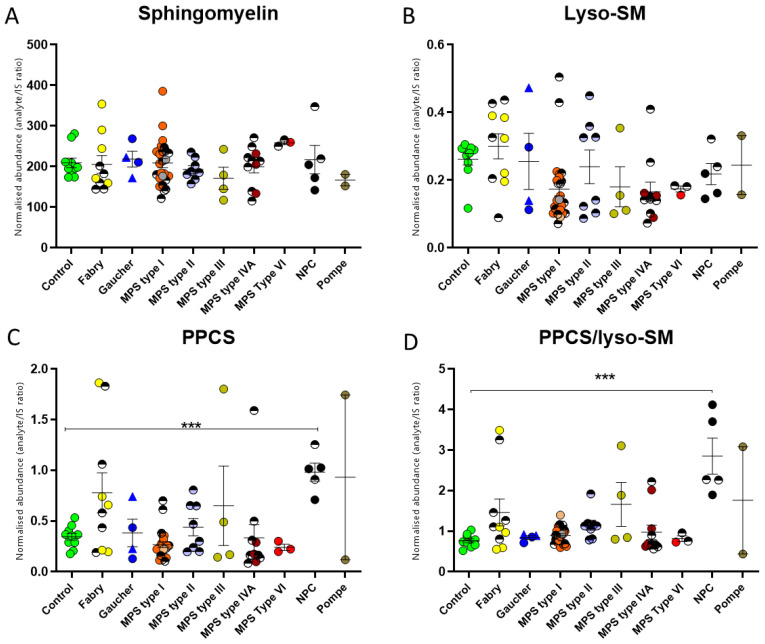
Levels of NPC biomarkers in a cohort of patients with lysosomal storage diseases. (**A**) Levels of total sphingomyelin in the LSD groups are similar to those seen in control samples. (**B**) Lyso-sphingomyelin levels are similar to those seen in the control cohort, with some outlier patients. (**C**) Total PPCS is significantly elevated in NPC but shows outliers in other disorders. (**D**) Ratioing of PPCS to lyso-sphingomyelin improves specificity for NPC relative to other LSDs. Patients on treatment are represented by half-shaded circles. Gaucher type I is indicated by a triangle symbol, and type III, by a circle. The untreated MPS type I group circles are coloured as follows: Hurler in orange, Hurler–Scheie in grey, and Scheie in pink. *** significance of *p* ≤ 0.001.

Sphingomyelin, lyso-sphingomyelin, and “lyso-sphingomyelin 509”, now identified as N-palmitoyl-O-phosphocholineserine [9] (PPCS), have all been reported as biomarkers for NPC [16,17]. Hence, we also included these biomarkers within our multiplex panel assay to see how specific they are for NPC relative to the other LSDs. No overall specific disease group changes were observed for sphingomyelin or lyso-sphingomyelin (Figure 5A,B), although some individuals within those groups did have levels higher than controls, as was the case of two FD patients, one MPS III patient, and one MPS IVA patient. Conversely, the levels of PPCS were increased significantly in the NPC but in no other disease group (Figure 5C). Kuchar et al. have demonstrated that the use of both PPCS and lyso-sphingomyelin helps discriminate NPC [17]. Ratioing PPCS to lyso-sphingomyelin shows that NPC samples have the highest (3-fold median) increased ratio (Figure 5D); however, similarly to SM and lyso-SM, some individuals within the other disease groups also have elevated levels. Therefore, these markers are not specific for NPC [18].

## 3. Discussion

Ordinarily, biomarkers for LSDs are tested against a relevant control population. We developed a multiplexed panel assay to assess the levels of known biomarkers in a cohort of patients with LSDs to better understand their specificity. Using this approach, we uncovered some novel findings regarding GSL metabolism in LSDs. In the case of the glycosphingolipidoses, typically, the glycosphingolipid substrate of the affected enzyme accumulates. However, we have shown here that the accumulation of these substrates can also occur in other disorders; e.g., whilst it is known that GlcCer accumulates in GD, some of the patients with Fabry, Hurler, and NPC disease also have increased GlcCer levels. Indeed, some of the NPC patients have GlcCer levels in the range of the Gaucher disease patients. A more detailed analysis, however, of the individual GlcCer species demonstrates that the GlcCer profile of the NPC patients is different from the controls and from those with GD. Specifically, fewer C16 GlcCer and longer-chain C24 GlcCer species are observed in NPC. GlcCer is hydrolysed to ceramide by glucocerebrosidase (GBA), of which there are two forms; one is lysosomal (GBA1) and typically is affected in GD disease, and the other (GBA2) is non-lysosomal. Previous work has shown that GBA acts as a trans-glucosidase, transferring glucose between GlcCer and cholesterol to create glucosylated cholesterol, which is elevated in GD and NPC [19]. This is thought to occur mainly from GBA2 activity. GBA2 is known to regulate endo-lysosomal function in NPC [20], and it may be attempting to modulate the accumulated cholesterol levels through glucosylation. This would reduce the hydrophobicity of the cholesterol compound, thereby promoting its removal from the cell. This could explain the upregulation of GlcCer in NPC, where it acts as the glucose donor; the disparity in the isoforms of GlcCer between GD and NPC could be indicative of the GlcCer origin being either lysosomal in GD or endo-lysosomal in NPC, where the common C16 isoform may be the primary donor/substrate for GBA2 cholesterol glucosylation.

Improved specificity for NPC detection can be achieved by looking at the ratio of the C24:1 to C16 isoform of GlcCer (Figure 3D). However, whilst the ratio in the NPC patients is distinct from the controls, elevated levels are not completely specific for NPC, with an overlap seen with levels in some FD and MPS patient samples, although they have lower levels than most NPC samples. Whilst this reduces the specificity of the ratioed species for NPC, FD and MPS are clinically very different, and the disorders could be distinguished further using their disease symptom phenotype.

We have shown previously that changes in the ratio of GlcCer to CDH C24:2-OH occur in NPC [1]. Levels of GlcCer/CDH C24:2-OH and PPCS/lyso-SM were analysed in a cohort of patients with known and unknown neurodevelopmental disease. GlcCer/CDH C24:2-OH had 100% sensitivity and specificity for NPC, and for PPCS/lyso-SM, 62.5% sensitivity and 90.91% specificity. Comparison of these published results with the current study, which looked at these biomarkers in a cohort of patients with LSD disorders, shows that the PPCS/lyso-SM ratio for NPC is more sensitive (92%) but less specific (63%) and that the GlcCer/CDH C24:2-OH ratio is slightly more discriminatory when looked at in an LSD cohort, with 96% sensitivity and 83% specificity.

Plasma chitotriosidase (CT) is routinely used as a surrogate marker for GD [21]. In practice, serum CT levels are hard to determine and interpret, as 6% of the population lack chitotriosidase activity [22]. Recently, there has been increased interest in the use of lyso-Gb1 as a primary biomarker for diagnosis and monitoring of the severity of GD disease [23,24,25]. We observed very high levels of lyso-Gb1 and lyso-Gb3, as expected, in the GD and FD samples, respectively. However, we also saw moderate increases in individual patients with other LSDs. These changes are likely to indicate a degree of lysosomal impairment rather than any effect on the GSL degradation pathway. Elevated GlcCer was observed in both the GD and NPC disease groups (Figure 2A); however, whilst there was also an accompanying elevation of lyso-Gb1 in the GD group, this was not evident for NPC (Figure 4A). This suggests that the elevated GlcCer in NPC may not have originated from the lysosome, where it would ordinarily be exposed to acid ceramidase and deacylated, and instead may have arisen from extra-lysosomal GlcCer that would be acted on by GBA2.

Analysis of the samples from MPS type III patients revealed a small, although significant, increase in lyso-Gb3. This was not unique to this group of patients, with some individuals in the MPS II group also having elevated lyso-Gb3 (Figure 4C). All the MPS II patients included in this study have the neuronopathic form of the disease and were on treatment at the time. Further longitudinal monitoring studies need to be performed in the future to ascertain whether the normal levels of lyso-Gb3 in some of these patients could be related to treatment response. This corroborates the findings of Baydakova et al. (2020), who observed increased DBS lyso-Gb3 in neuronopathic forms of MPS (including MPS III) [26].

Low levels of lyso-CDH have been observed previously in GD and NPC plasma [16,25]. Our method detected an increase of 5.9-fold in the GD patients and 2-fold in the FD patients relative to controls, although there was no corresponding increase in the DBS CDH for either disorder. A potential explanation for this is the accumulation or “backing up” of the GSL degradation pathway prior to the block, causing the increase in lyso-CDH. In FD, the lyso-CDH observed is likely to be lyso-Gb2, as urinary Gb2 accumulation occurs in FD [11,12]. It is possible that we have, unlike other studies, been able to detect this increase in GD and FD samples due to the improved sensitivity of more recent generation mass spectrometers. In previous studies, lyso-CDH was used as an internal standard in mass-spectrometry-based biomarker assays for FD and GD [25,27,28,29]. Given our observations, caution should be exercised if lyso-Gb2 is to be used as an internal standard in the future.

In conclusion, our study demonstrates the utility of a biomarker panel approach for detecting glycosphingolipidoses and LSDs. This approach can also be used to distinguish between different subgroups. Whilst disease-specific biomarker levels can overlap with other similar disorders, the use of multiple biomarkers can help establish a diagnosis more promptly. Examination of a range of pathophysiologically relevant markers also sheds insight into the common downstream pathophysiological mechanism(s) leading to intracellular dysfunction in these disorders. Such biomarker panel strategies represent a simplified, fast, and accurate option to expedite the diagnostic process in neurometabolic and other rare disorders; they have translational potential and a role in accelerating the time to diagnosis and, ultimately, implementing targeted disease-specific treatments that are becoming increasingly available in the field.

## 4. Materials and Methods

### 4.1. Reagents and Standards

Methanol, acetonitrile, chloroform, and ethanol were from Fisher Chemicals. Formic acid and DMSO were from Sigma-Aldrich, Gillingham UK. All solvents were HPLC-grade, apart from formic acid, which was UPLC-grade. Water used was Milli-Q grade (Milipore, UK). Sphingolipid internal standards were obtained from Matreya LLC (State College, PA, USA): ^13^C_6_ glucosyl-sphingosine, N-glycinated lyso-ceramide trihexoside, N-omega-CD3-hexadecanoyl-glucopsychosine, N-omega-CD3-hexadecanoyl-lactosylceramide, and N-omega-CD3-octadecanoyl-ceramide trihexoside further details are available in Supplementary Table S2.

### 4.2. Samples

This study was approved by the National Research Ethics Service (NRES) in the UK (NRES Committee: London—Bloomsbury, REC reference: 13/LO/0168, IRAS project ID: 95005, Study of Inherited Metabolic Disease [SIMD]). Paediatric DBS samples were from Gaucher (*n* = 4), Fabry (*n* = 10 (4 on treatment)), Pompe (*n* = 2), MPS types I–VI (*n* = 52, (9 on treatment)), and NPC (*n* = 5, (2 on treatment)) patients. Diagnosis of these patients has been confirmed by genetic and biochemical enzyme testing, except in the case of NPC, where the diagnosis has been confirmed genetically. Patients were grouped according to their genetic diagnosis. MPS I patients were further stratified on the basis of their neurological assessment; i.e., Hurler patients have neurodegeneration, Hurler–Scheie patients have mild mental retardation, and individuals with Scheie disease are unaffected. In all cases, written informed consent was obtained. Samples were stored at −80 °C prior to analysis. Control blood spots (*n* = 10) were obtained from healthy volunteers. Further sample information is given in Supplementary Table S1.

### 4.3. Dried Blood Spot Sample Preparation

DBSs were prepared as described previously [1]. Briefly, one 6 mm blood spot was punched into a 1.5 mL Eppendorf tube. Then, 100 µL of extraction solution (45% acetonitrile: 30% chloroform: 10% DMSO: 10% ethanol: 5% water) containing internal standards was added to the spot and the tube left for 3 min at room temperature. The sample was then centrifuged at 13,000× *g* for 5 min until the punch was fully submerged, prior to sonication for 10 min in an ultrasonic water bath and centrifugation at 13,000× *g* for 5 min. Subsequently, 90 µL of the extract was transferred into a glass vial containing 90 µL of methanol. To control for instrument performance during the UPLC–MS/MS run, a pooled sample was created by combining equal volumes from each sample.

### 4.4. UPLC–MS/MS Analysis

A Waters Acquity ultra-performance liquid chromatograph (UPLC) coupled with a Xevo TQ-S triple quadrupole mass spectrometer (Waters Corp., Manchester, UK) was used in positive mode for the analysis. Compounds were separated on an ACQUITY UPLC 2.1 × 50 mm BEH C8 1.7-μm column (Waters Corp., UK). The pooled sample was injected at regular intervals during the run. For each injection, starting conditions were: 0.5 mL/min of 50% A (0.05% formic acid in Milli-Q water): 50% B (methanol). The column temperature was set to 40 °C. All UPLC–MS/MS method details have been described previously [1].

### 4.5. Data and Statistical Analysis

Mass spectrometry data were analysed using TargetLynx in MassLynx v 4.2 software (Waters Corp.). The relative abundance of each compound was obtained by ratioing its area to the corresponding internal standard area. A non-parametric Mann–Whitney test was used, and a *p* value < 0.05 considered significant.

## Data Availability

Data are contained within the article and Supplementary Materials.

## Supplementary Materials

The supporting information can be downloaded at: https://www.mdpi.com/article/10.3390/ijms241210177/s1.

## References

[1] Papandreou A., Doykov I., Spiewak J., Komarov N., Habermann S., Kurian M.A., Mills P.B., Mills K., Gissen P., Heywood W.E. (2022). Niemann-Pick type C disease as proof-of-concept for intelligent biomarker panel selection in neurometabolic disorders. Dev. Med. Child Neurol..

[2] Dinur T., Bauer P., Beetz C., Kramp G., Cozma C., Iurascu M.I., Becker-Cohen M., Istaiti M., Rolfs A., Zimran A. (2022). Gaucher Disease Diagnosis Using Lyso-Gb1 on Dry Blood Spot Samples: Time to Change the Paradigm?. Int. J. Mol. Sci..

[3] Malvagia S., Ferri L., Della Bona M., Borsini W., Cirami C.L., Dervishi E., Feriozzi S., Gasperini S., Motta S., Mignani R. (2021). Multicenter evaluation of use of dried blood spot compared to conventional plasma in measurements of globotriaosylsphingosine (LysoGb3) concentration in 104 Fabry patients. Clin. Chem. Lab. Med..

[4] Khaledi H., Gelb M.H. (2020). Tandem Mass Spectrometry Enzyme Assays for Multiplex Detection of 10-Mucopolysaccharidoses in Dried Blood Spots and Fibroblasts. Anal. Chem..

[5] Young E., Mills K., Morris P., Vellodi A., Lee P., Waldek S., Winchester B. (2005). Is globotriaosylceramide a useful biomarker in Fabry disease?. Acta Paed..

[6] Tang C., Jia X., Tang F., Liu S., Jiang X., Zhao X., Sheng H., Peng M., Liu L., Huang Y. (2021). Detection of glucosylsphingosine in dried blood spots for diagnosis of Gaucher disease by LC-MS/MS. Clin. Biochem..

[7] Viana G.M., Priestman D.A., Platt F.M., Khan S., Tomatsu S., Pshezhetsky A.V. (2020). Brain Pathology in Mucopolysaccharidoses (MPS) Patients with Neurological Forms. J. Clin. Med..

[8] Soldati C., Lopez-Fabuel I., Wanderlingh L.G., Garcia-Macia M., Monfregola J., Esposito A., Napolitano G., Guevara-Ferrer M., Scotto Rosato A., Krogsaeter E.K. (2021). Repurposing of tamoxifen ameliorates CLN3 and CLN7 disease phenotype. EMBO Mol. Med..

[9] Sidhu R., Mondjinou Y., Qian M., Song H., Kumar A.B., Hong X., Hsu F.F., Dietzen D.J., Yanjanin N.M., Porter F.D. (2019). N-acyl-O-phosphocholineserines: Structures of a novel class of lipids that are biomarkers for Niemann-Pick C1 disease. J. Lipid Res..

[10] Kasapkara Ç.S., Ceylan A.C., Yılmaz D., Kıreker Köylü O., Yürek B., Civelek Ürey B., Gündüz M. (2022). CLN3-Associated NCL Case with a Preliminary Diagnosis of Niemann Pick Type C. Mol. Syndromol..

[11] Boutin M., Menkovic I., Martineau T., Vaillancourt-Lavigueur V., Toupin A., Auray-Blais C. (2017). Separation and Analysis of Lactosylceramide, Galabiosylceramide, and Globotriaosylceramide by LC-MS/MS in Urine of Fabry Disease Patients. Anal. Chem..

[12] Heywood W.E., Doykov I., Spiewak J., Hallqvist J., Mills K., Nowak A. (2019). Global glycosphingolipid analysis in urine and plasma of female Fabry disease patients. Biochim. Biophys. Acta Mol. Basis Dis..

[13] Mills K., Morris P., Lee P., Vellodi A., Waldek S., Young E., Winchester B. (2005). Measurement of urinary CDH and CTH by tandem mass spectrometry in patients hemizygous and heterozygous for Fabry disease. J. Inherit. Metab. Dis..

[14] Carnicer-Caceres C., Arranz-Amo J.A., Cea-Arestin C., Camprodon-Gomez M., Moreno-Martinez D., Lucas-Del-Pozo S., Molto-Abad M., Tigri-Santina A., Agraz-Pamplona I., Rodriguez-Palomares J.F. (2021). Biomarkers in Fabry Disease. Implications for Clinical Diagnosis and Follow-up. J. Clin. Med..

[15] Kusano E., Saito O., Akimoto T., Asano Y. (2014). Fabry disease: Experience of screening dialysis patients for Fabry disease. Clin. Exp. Nephrol..

[16] Giese A.K., Mascher H., Grittner U., Eichler S., Kramp G., Lukas J., te Vruchte D., Al Eisa N., Cortina-Borja M., Porter F.D. (2015). A novel, highly sensitive and specific biomarker for Niemann-Pick type C1 disease. Orphanet J. Rare Dis..

[17] Kuchar L., Sikora J., Gulinello M.E., Poupetova H., Lugowska A., Malinova V., Jahnova H., Asfaw B., Ledvinova J. (2017). Quantitation of plasmatic lysosphingomyelin and lysosphingomyelin-509 for differential screening of Niemann-Pick A/B and C diseases. Anal. Biochem..

[18] Sitarska D., Lugowska A. (2019). Laboratory diagnosis of the Niemann-Pick type C disease: An inherited neurodegenerative disorder of cholesterol metabolism. Metab. Brain Dis..

[19] Marques A.R., Mirzaian M., Akiyama H., Wisse P., Ferraz M.J., Gaspar P., Ghauharali-van der Vlugt K., Meijer R., Giraldo P., Alfonso P. (2016). Glucosylated cholesterol in mammalian cells and tissues: Formation and degradation by multiple cellular beta-glucosidases. J. Lipid Res..

[20] Wheeler S., Haberkant P., Bhardwaj M., Tongue P., Ferraz M.J., Halter D., Sprong H., Schmid R., Aerts J., Sullo N. (2019). Cytosolic glucosylceramide regulates endolysosomal function in Niemann-Pick type C disease. Neurobiol. Dis..

[21] Hollak C.E., van Weely S., van Oers M.H., Aerts J.M. (1994). Marked elevation of plasma chitotriosidase activity. A novel hallmark of Gaucher disease. J. Clin. Investig..

[22] Boot R.G., Renkema G.H., Verhoek M., Strijland A., Bliek J., de Meulemeester T.M., Mannens M.M., Aerts J.M. (1998). The human chitotriosidase gene. Nature of inherited enzyme deficiency. J. Biol. Chem..

[23] Murugesan V., Chuang W.L., Liu J., Lischuk A., Kacena K., Lin H., Pastores G.M., Yang R., Keutzer J., Zhang K. (2016). Glucosylsphingosine is a key biomarker of Gaucher disease. Am. J. Hematol..

[24] Dekker N., van Dussen L., Hollak C.E., Overkleeft H., Scheij S., Ghauharali K., van Breemen M.J., Ferraz M.J., Groener J.E., Maas M. (2011). Elevated plasma glucosylsphingosine in Gaucher disease: Relation to phenotype, storage cell markers, and therapeutic response. Blood.

[25] Rolfs A., Giese A.K., Grittner U., Mascher D., Elstein D., Zimran A., Bottcher T., Lukas J., Hubner R., Golnitz U. (2013). Glucosylsphingosine is a highly sensitive and specific biomarker for primary diagnostic and follow-up monitoring in Gaucher disease in a non-Jewish, Caucasian cohort of Gaucher disease patients. PLoS ONE.

[26] Baydakova G., Ilyushkina A., Gaffke L., Pierzynowska K., Bychkov I., Lugowska A., Wegrzyn G., Tylki-Szymanska A., Zakharova E. (2020). Elevated LysoGb3 Concentration in the Neuronopathic Forms of Mucopolysaccharidoses. Diagnostics.

[27] Johnson B., Mascher H., Mascher D., Legnini E., Hung C.Y., Dajnoki A., Chien Y.H., Marodi L., Hwu W.L., Bodamer O.A. (2013). Analysis of lyso-globotriaosylsphingosine in dried blood spots. Ann. Lab. Med..

[28] Lukas J., Scalia S., Eichler S., Pockrandt A.M., Dehn N., Cozma C., Giese A.K., Rolfs A. (2016). Functional and Clinical Consequences of Novel alpha-Galactosidase A Mutations in Fabry Disease. Hum. Mutat..

[29] Lukas J., Cozma C., Yang F., Kramp G., Meyer A., Nesslauer A.M., Eichler S., Bottcher T., Witt M., Brauer A.U. (2017). Glucosylsphingosine Causes Hematological and Visceral Changes in Mice-Evidence for a Pathophysiological Role in Gaucher Disease. Int. J. Mol. Sci..

