# Supplementary material for: New Perspectives in Dried Blood Spot Biomarkers for Lysosomal Storage Diseases
Source: Int J Mol Sci. 2023 Jun 15;24(12):10177. doi: 10.3390/ijms241210177 (PMC10299042; doi:10.3390/ijms241210177)
Supplement: Supplementary file 1 [file ijms-24-10177-s001.zip › Supporting infomation IS and transition table.pdf]

Table S2. Mass transitions and conditions for all internal standards and compounds used in this study. CMH = ceramide monohexoside, CDH ceramide dihexoside, SM = sphingomyelin.

All standards were purchased from Matreya LLC

**Run 1**				
**Compound**	**Internal Standard**	**Transition**	**Cone Volt.**	**Col.Energy**
Lyso-Gb1-28	$^{13}C_6$ Glucosyl-sphingosine	434.37 > 236.32	92	12
Lyso-Gb1-12	$^{13}C_6$ Glucosyl-sphingosine	450.44 > 252.38	92	12
Lyso-Gb1-2	$^{13}C_6$ Glucosyl-sphingosine	460.39 > 262.33	92	12
Lyso-Gb1	$^{13}C_6$ Glucosyl-sphingosine	462.41 > 264.35	92	12
Lyso-Gb1	$^{13}C_6$ Glucosyl-sphingosine	462.41 > 282.36	92	16
$^{13}C_6$ Glucosyl-sphingosine	n/a	468.41 > 264.35	92	20
$^{13}C_6$ Glucosyl-sphingosine	n/a	468.41 > 282.36	92	20
Lyso-Gb1+14	$^{13}C_6$ Glucosyl-sphingosine	476.4 > 278.34	92	12
Lyso-Gb1+16	$^{13}C_6$ Glucosyl-sphingosine	478.4 > 280.34	92	12
Lyso-Gb1+34	$^{13}C_6$ Glucosyl-sphingosine	496.41 > 298.36	92	12
Lyso-Gb1+50	$^{13}C_6$ Glucosyl-sphingosine	512.4 > 314.34	92	12
Lyso-CDH-28	$^{13}C_6$ Glucosyl-sphingosine	596.43 > 236.32	92	12
Lyso-CDH-12	$^{13}C_6$ Glucosyl-sphingosine	612.49 > 252.38	92	12
Lyso-CDH-2	$^{13}C_6$ Glucosyl-sphingosine	622.44 > 262.33	92	12
Lyso-CDH	$^{13}C_6$ Glucosyl-sphingosine	624.46 > 264.35	92	12
Lyso-CDH	$^{13}C_6$ Glucosyl-sphingosine	624.46 > 282.36	92	16
Lyso-CDH+14	$^{13}C_6$ Glucosyl-sphingosine	638.45 > 278.34	92	12
Lyso-CDH+16	$^{13}C_6$ Glucosyl-sphingosine	640.46 > 280.34	92	12
Lyso-CDH+34	$^{13}C_6$ Glucosyl-sphingosine	658.47 > 298.36	92	12
Lyso-CDH+50	$^{13}C_6$ Glucosyl-sphingosine	674.45 > 314.34	92	12
**Compound**	**Internal Standard**	**Transition**	**Cone Volt.**	**Col.Energy**
CMH_C16:0	N-omega-CD3-Octadecanoyl-ceramide trihexoside	722.48 > 560.51	125	42
CMH_C18:0	N-omega-CD3-Octadecanoyl-ceramide trihexoside	750.53 > 588.56	125	42
CMH_C20:0	N-omega-CD3-Octadecanoyl-ceramide trihexoside	778.59 > 616.62	125	42
CMH_C22:1	N-omega-CD3-Octadecanoyl-ceramide trihexoside	804.62 > 642.65	125	42
CMH_C22:0	N-omega-CD3-Octadecanoyl-ceramide trihexoside	806.64 > 644.67	125	42
CMH_C24:2	N-omega-CD3-Octadecanoyl-ceramide trihexoside	830.66 > 668.69	125	42
CMH_C24:1	N-omega-CD3-Octadecanoyl-ceramide trihexoside	832.68 > 670.71	125	42
CMH_C24:0	N-omega-CD3-Octadecanoyl-ceramide trihexoside	834.69 > 672.72	125	42
CMH_C24:2-OH	N-omega-CD3-Octadecanoyl-ceramide trihexoside	846.65 > 684.68	125	42
CMH_C24:1-OH	N-omega-CD3-Octadecanoyl-ceramide trihexoside	848.67 > 686.70	125	42
CMH_C24:0-OH	N-omega-CD3-Octadecanoyl-ceramide trihexoside	850.69 > 688.72	125	42
CMH_C26:0	N-omega-CD3-Octadecanoyl-ceramide trihexoside	862.74 > 700.78	125	42

N-omega-CD3-Octadecanoyl-ceramide trihexoside	n/a	1077.84 > 915.91	108	56
Gb4_C16:0	N-omega-CD3-Octadecanoyl-ceramide trihexoside	1249.44 > 1046.46	125	52
Gb4_C18:1	N-omega-CD3-Octadecanoyl-ceramide trihexoside	1275.47 > 1072.50	125	52
Gb4_C18:0	N-omega-CD3-Octadecanoyl-ceramide trihexoside	1277.49 > 1074.51	125	52
Gb4_C20:1	N-omega-CD3-Octadecanoyl-ceramide trihexoside	1303.53 > 1100.55	125	52
Gb4_C20:0	N-omega-CD3-Octadecanoyl-ceramide trihexoside	1305.54 > 1102.56	125	52
Gb4_C22:1	N-omega-CD3-Octadecanoyl-ceramide trihexoside	1331.58 > 1128.60	125	52
Gb4_C22:0	N-omega-CD3-Octadecanoyl-ceramide trihexoside	1333.60 > 1130.62	125	52
Gb4_C24:2	N-omega-CD3-Octadecanoyl-ceramide trihexoside	1357.62 > 1154.64	125	52
Gb4_C24:1	N-omega-CD3-Octadecanoyl-ceramide trihexoside	1359.63 > 1156.66	125	52
Gb4_C24:0	N-omega-CD3-Octadecanoyl-ceramide trihexoside	1361.65 > 1158.67	125	52
Gb4_C24:2-OH	N-omega-CD3-Octadecanoyl-ceramide trihexoside	1373.61 > 1170.63	125	52
Gb4_C24:1-OH	N-omega-CD3-Octadecanoyl-ceramide trihexoside	1375.63 > 1172.65	125	52
Gb4_C24:0-OH	N-omega-CD3-Octadecanoyl-ceramide trihexoside	1377.64 > 1174.67	125	52
Gb4_C26:1	N-omega-CD3-Octadecanoyl-ceramide trihexoside	1387.69 > 1184.71	125	52
Gb4_C26:0	N-omega-CD3-Octadecanoyl-ceramide trihexoside	1389.70 > 1186.72	125	52

Run 2

**Compound**	**Internal Standard**	**Transition**	**Cone Volt.**	**Col.Energy**
Lyso SM -28	$^{13}C_6$ Glucosyl-sphingosine	437.60 > 184.07	52	20
Lyso SM -12	$^{13}C_6$ Glucosyl-sphingosine	453.66 > 184.07	52	20
Lyso SM -2	$^{13}C_6$ Glucosyl-sphingosine	463.61 > 184.07	52	20
Lyso SM	$^{13}C_6$ Glucosyl-sphingosine	465.63 > 184.07	52	20
Lyso SM	$^{13}C_6$ Glucosyl-sphingosine	465.63 > 264.35	52	20
$^{13}C_6$ Glucosyl-sphingosine	n/a	468.41 > 264.35	92	20
$^{13}C_6$ Glucosyl-sphingosine	n/a	468.41 > 282.36	92	20
Lyso SM 14	$^{13}C_6$ Glucosyl-sphingosine	479.62 > 184.07	52	20
Lyso SM 16	$^{13}C_6$ Glucosyl-sphingosine	481.62 > 184.07	52	20
Lyso SM 34	$^{13}C_6$ Glucosyl-sphingosine	499.64 > 184.07	52	20
Lyso SM 50	$^{13}C_6$ Glucosyl-sphingosine	515.62 > 332.56	52	20
Lyso-Gb3-28	N-Glycinated lyso-ceramide trihexoside	758.37 > 236.32	72	22
Lyso-Gb3-12	N-Glycinated lyso-ceramide trihexoside	774.44 > 252.38	72	22
Lyso-Gb3-2	N-Glycinated lyso-ceramide trihexoside	784.39 > 262.33	72	22
Lyso-Gb3	N-Glycinated lyso-ceramide trihexoside	786.41 > 264.35	72	22
Lyso-Gb3	N-Glycinated lyso-ceramide trihexoside	786.41 > 282.36	72	28
Lyso-Gb3+14	N-Glycinated lyso-ceramide trihexoside	800.40 > 278.34	72	22
Lyso-Gb3+16	N-Glycinated lyso-ceramide trihexoside	802.40 > 280.34	72	22

Lyso-Gb3+34	N-Glycinated lyso-ceramide trihexoside	820.41 > 298.36	72	22
Lyso-Gb3+50	N-Glycinated lyso-ceramide trihexoside	836.40 > 314.34	72	22
N-Glycinated lyso-ceramide trihexo-side	n/a	843.64 > 264.43	48	48
N-Glycinated lyso-ceramide trihexo-side	n/a	843.64 > 339.45	48	26
**Compound**	**Internal Standard**	**Transition**	**Cone Volt.**	**Col.Energy**
Lyso SM 509	$^{13}C_6$ Glucosyl-sphingosine	509.39 > 184.07	52	30
Lyso SM 509	$^{13}C_6$ Glucosyl-sphingosine	509.39 > 326.33	52	20
CDH_C16:1	N-omega-CD3-Octadecanoyl-ceramide trihexoside	882.22 > 720.25	124	57
CDH_C16:0	N-omega-CD3-Octadecanoyl-ceramide trihexoside	884.24 > 722.27	124	57
CDH_C18:1	N-omega-CD3-Octadecanoyl-ceramide trihexoside	910.27 > 748.30	124	57
CDH_C18:0	N-omega-CD3-Octadecanoyl-ceramide trihexoside	912.29 > 750.32	124	57
CDH_C20:1	N-omega-CD3-Octadecanoyl-ceramide trihexoside	938.33 > 776.36	124	57
CDH_C20:0	N-omega-CD3-Octadecanoyl-ceramide trihexoside	940.34 > 778.37	124	57
CDH_C22:1	N-omega-CD3-Octadecanoyl-ceramide trihexoside	966.38 > 804.41	124	57
CDH_C22:0	N-omega-CD3-Octadecanoyl-ceramide trihexoside	968.40 > 806.43	124	57
CDH_C24:3	N-omega-CD3-Octadecanoyl-ceramide trihexoside	990.40 > 828.43	124	57
CDH_C24:2	N-omega-CD3-Octadecanoyl-ceramide trihexoside	992.42 > 830.45	124	57
CDH_C24:1	N-omega-CD3-Octadecanoyl-ceramide trihexoside	994.43 > 832.46	124	57
CDH_C24:0	N-omega-CD3-Octadecanoyl-ceramide trihexoside	996.45 > 834.48	124	57
CDH_C24:3 -OH	N-omega-CD3-Octadecanoyl-ceramide trihexoside	1006.40 > 844.43	124	57
CDH_C24:2-OH	N-omega-CD3-Octadecanoyl-ceramide trihexoside	1008.41 > 846.44	124	57
CDH_C24:1-OH	N-omega-CD3-Octadecanoyl-ceramide trihexoside	1010.43 > 848.46	124	57
CDH_C24:0-OH	N-omega-CD3-Octadecanoyl-ceramide trihexoside	1012.44 > 850.47	124	57
CDH_C26:3	N-omega-CD3-Octadecanoyl-ceramide trihexoside	1018.46 > 856.48	124	57
CDH_C26:2	N-omega-CD3-Octadecanoyl-ceramide trihexoside	1020.47 > 858.50	124	57
CDH_C26:1	N-omega-CD3-Octadecanoyl-ceramide trihexoside	1022.49 > 860.52	124	57
CDH_C26:0	N-omega-CD3-Octadecanoyl-ceramide trihexoside	1024.50 > 862.53	124	57
CDH_C26:3 -OH	N-omega-CD3-Octadecanoyl-ceramide trihexoside	1034.45 > 872.48	124	57
CDH_C26:2-OH	N-omega-CD3-Octadecanoyl-ceramide trihexoside	1036.46 > 874.49	124	57
CDH_C26:1-OH	N-omega-CD3-Octadecanoyl-ceramide trihexoside	1038.48 > 876.51	124	57
CDH_C26:0-OH	N-omega-CD3-Octadecanoyl-ceramide trihexoside	1040.50 > 878.53	124	57
N-omega-CD3-Octadecanoyl-ceramide trihexoside	n/a	1077.84 > 915.91	108	56
**Run 3**				

Compound	Internal Standard	Transition	Cone Volt.	Col.Energy
SM_C16:1	N-Heptadecanoyl-sphingo-sylphosphorylcholine	723.43 > 540.41	90	40
SM_C16:0	N-Heptadecanoyl-sphingo-sylphosphorylcholine	725.44 > 542.43	90	40
N-Heptadecanoyl-sphingo-sylphosphorylcholine	n/a	739.46 > 556.44	90	40
SM_C18:1	N-Heptadecanoyl-sphingo-sylphosphorylcholine	751.46 > 568.44	90	40
SM_C18:0	N-Heptadecanoyl-sphingo-sylphosphorylcholine	753.48 > 570.46	90	40
SM_C20:1	N-Heptadecanoyl-sphingo-sylphosphorylcholine	779.49 > 596.47	90	40
SM_C20:0	N-Heptadecanoyl-sphingo-sylphosphorylcholine	781.51 > 598.49	90	40
SM_C22:1	N-Heptadecanoyl-sphingo-sylphosphorylcholine	807.52 > 624.51	90	45
SM_C22:0	N-Heptadecanoyl-sphingo-sylphosphorylcholine	809.54 > 626.52	90	45
SM_C24:2	N-Heptadecanoyl-sphingo-sylphosphorylcholine	833.54 > 650.52	90	45
SM_C24:1	N-Heptadecanoyl-sphingo-sylphosphorylcholine	835.55 > 652.54	90	45
SM_C24:0	N-Heptadecanoyl-sphingo-sylphosphorylcholine	837.57 > 654.55	90	45
SM_C26:0	N-Heptadecanoyl-sphingo-sylphosphorylcholine	865.60 > 682.58	90	45
Gb3_C16:0	N-omega-CD3-Octadecanoyl-ceramide trihexoside	1046.63 > 884.66	124	64
Gb3_C18:0	N-omega-CD3-Octadecanoyl-ceramide trihexoside	1074.68 > 912.71	124	64
N-omega-CD3-Octadecanoyl-ceramide trihexoside	n/a	1077.84 > 915.91	108	56
Gb3_C20:0	N-omega-CD3-Octadecanoyl-ceramide trihexoside	1102.74 > 940.77	124	64
Gb3_C22:1	N-omega-CD3-Octadecanoyl-ceramide trihexoside	1128.77 > 966.80	124	64
Gb3_C22:0	N-omega-CD3-Octadecanoyl-ceramide trihexoside	1130.79 > 968.82	124	64
Gb3_C24:2	N-omega-CD3-Octadecanoyl-ceramide trihexoside	1154.81 > 992.84	124	64
Gb3_C24:1	N-omega-CD3-Octadecanoyl-ceramide trihexoside	1156.83 > 994.86	124	64
Gb3_C24:0	N-omega-CD3-Octadecanoyl-ceramide trihexoside	1158.84 > 996.87	124	64
Gb3_C24:2-OH	N-omega-CD3-Octadecanoyl-ceramide trihexoside	1170.81 > 1008.84	124	64
Gb3_C24:1-OH	N-omega-CD3-Octadecanoyl-ceramide trihexoside	1172.82 > 1010.85	124	64
Gb3_C24:0-OH	N-omega-CD3-Octadecanoyl-ceramide trihexoside	1174.84 > 1012.87	124	64
Gb3_C26:0	N-omega-CD3-Octadecanoyl-ceramide trihexoside	1186.90 > 1024.93	124	64
